# Psychosis in Alzheimer’s Disease

**DOI:** 10.1002/alz.083393

**Published:** 2025-01-09

**Authors:** Clive G Ballard

**Affiliations:** ^1^ King’s College London, London, United Kingdom; College of Medicine and Health, University of Exeter, Exeter United Kingdom

## Abstract

Psychosis is a common and distressing disorder in people with Alzheimer disease, associated with a poor clinical prognosis, an increased risk of institutionalization and for which there are no approved treatments. New approaches to diagnosis and symptom assessment and treatment are beginning to move the field forward, including the emergence of psychosis at the pre‐clinical or even pre‐cognitive impairment stages of disease in some individuals. The Alzheimer’s Association International Society to Advance Alzheimer’s Research and Treatment (ISTAART) research criteria for psychosis in neurodegenerative disease, and the ISTAART criteria for mild behavioural impairment are examples of recent developments. New genomic, neuroimaging, post‐mortem and neurobiology studies are beginning to refine our mechanistic understanding and providing novel opportunities for drug discovery, drug repurposing and potentially for better approaches to precision medicine. Emerging potential therapies include the 5HT2A inverse agonist pimavanserin, which is already licensed in the US for the treatment of Parkinson’s disease, escitalopram, muscarinic agonists and cannabidiol. Emerging data also highlight opportunities to optimize and develop more targeted psychological therapies for people with Alzheimer’s disease psychosis. The treatment of psychosis also remains a major challenge in synuclein dementias where psychosis is more frequent and more persistent, and where many patients experience severe sensitivity reactions to antipsychotic medications. There is very little work examining the mechanisms or treatment of psychosis in people with vascular dementia, which remains a major unmet need. Although the assessment and management of psychosis in people with dementia remain challenging, improved diagnosis, evolving mechanistic understanding and an increased focus on new treatment studies are providing direction and new opportunities to the field and to people with Alzheimer’s disease.

